# Transcriptional and functional complexity of *Shank3* provides a molecular framework to understand the phenotypic heterogeneity of *SHANK3* causing autism and *Shank3* mutant mice

**DOI:** 10.1186/2040-2392-5-30

**Published:** 2014-04-25

**Authors:** Xiaoming Wang, Qiong Xu, Alexandra L Bey, Yoonji Lee, Yong-hui Jiang

**Affiliations:** 1 Division of Medical Genetics, Department of Pediatrics, Duke University School of Medicine, 27710 Durham, NC, USA; 2 Department of Neurobiology, Duke University School of Medicine, 27710 Durham, NC, USA; 3 Department of Child Health Care, Children’s Hospital of Fudan University, 201102 Shanghai, China

**Keywords:** Activity-dependent gene regulation, Alternative splicing, Autism spectrum disorder, Phenotypic heterogeneity, Shank3 isoform

## Abstract

**Background:**

Considerable clinical heterogeneity has been well documented amongst individuals with autism spectrum disorders (ASD). However, little is known about the biological mechanisms underlying phenotypic diversity. Genetic studies have established a strong causal relationship between ASD and molecular defects in the *SHANK3* gene. Individuals with various defects of *SHANK3* display considerable clinical heterogeneity. Different lines of *Shank3* mutant mice with deletions of different portions of coding exons have been reported recently. Variable synaptic and behavioral phenotypes have been reported in these mice, which makes the interpretations for these data complicated without the full knowledge of the complexity of the *Shank3* transcript structure.

**Methods:**

We systematically examined alternative splicing and isoform-specific expression of *Shank3* across different brain regions and developmental stages by regular RT-PCR, quantitative real time RT-PCR (q-PCR), and western blot. With these techniques, we also investigated the effects of neuronal activity and epigenetic modulation on alternative splicing and isoform-specific expression of *Shank3*. We explored the localization and influence on dendritic spine development of different Shank3 isoforms in cultured hippocampal neurons by cellular imaging.

**Results:**

The *Shank3* gene displayed an extensive array of mRNA and protein isoforms resulting from the combination of multiple intragenic promoters and extensive alternative splicing of coding exons in the mouse brain. The isoform-specific expression and alternative splicing of *Shank3* were brain-region/cell-type specific, developmentally regulated, activity-dependent, and involved epigenetic regulation. Different subcellular distribution and differential effects on dendritic spine morphology were observed for different Shank3 isoforms.

**Conclusions:**

Our results indicate a complex transcriptional regulation of *Shank3* in mouse brains. Our analysis of select *Shank3* isoforms in cultured neurons suggests that different *Shank3* isoforms have distinct functions. Therefore, the different types of *SHANK3* mutations found in patients with ASD and different exonic deletions of *Shank3* in mutant mice are predicted to disrupt selective isoforms and result in distinct dysfunctions at the synapse with possible differential effects on behavior. Our comprehensive data on *Shank3* transcriptional regulation thus provides an essential molecular framework to understand the phenotypic diversity in *SHANK3* causing ASD and *Shank3* mutant mice.

## Background

Shank3/ProSAP2 is one of three members of the Shank/ProSAP family of proteins which contain five conserved protein domains – an ankyrin repeat (ANK), a Src homology 3 (SH3), a PSD-95/Discs large/ZO-1 (PDZ), a proline-rich region containing homer- and cortactin-binding sites (Pro), and a sterile alpha motif (SAM) [1-3]. Shank proteins localize in the postsynaptic density (PSD) of excitatory synapses where they function as master scaffolding proteins by interacting directly or indirectly with various proteins, including major types of glutamate receptors – NMDARs, AMPARs, and mGluRs – via different domains [1,2,4-8].

Human genetic studies strongly support the notion that molecular defects of *SHANK3* contribute to autism spectrum disorders (ASD). In humans, *SHANK3* maps to the critical region of the 22q13.3 deletion syndrome (Phelan-McDermid syndrome; PMS), in which autistic behaviors are an important feature [9]. In addition, *de novo* sequence variants including missense, frame-shift, and splice site mutations across all coding exons of *SHANK3* have been identified in ~0.5% of ASD patients with variable clinical presentations [10-14]. Interestingly, *SHANK3* mutations were also reported in patients with childhood-onset schizophrenia and intellectual disability [15]. In the cases with point mutations or small deletions of *SHANK3*, it was noted that clinical features are also quite variable [8]. *Shank3* mutant mice with deletions of exons encoding ANK, SH3, and PDZ domains and proline-rich region have been reported [16-20]. These mutant mice shared some similarities but also have significant differences in synaptic defects and behavioral abnormalities. The interpretations for the data from different lines of mutant mice were complicated at the time by the lack of clear understanding of the complexity of *Shank3* transcript structure. It was believed that different lines of mutant mice only disrupted a select set of Shank3 isoforms. These observations then demand more knowledge of transcriptional regulation of *Shank3* in the brain, and pose an interesting question about the molecular basis underlying the clinical heterogeneity in human patients with *SHANK3* defects and the variability in different *Shank3* mutant mice.

*SHANK3* undergoes complex transcriptional regulation [8,21-24]. We and others have determined that *Shank3* displays multiple intragenic promoters and alternative splicing of coding exons in both mice and humans [12,18,23,25,26]. The combination of multiple promoters and alternative splicing is predicted to produce an extensive array of mRNA and protein isoforms, but this has not been fully characterized. With the information of presumptive *SHANK3* isoforms, point mutations or small exonic deletions of *SHANK3* found in ASD patients are predicted to affect selective isoforms of *SHANK3*. Since each SHANK3 isoform contains a distinct combination of the five different protein-protein interaction domains, each isoform may have a different function at the synapse. One interesting hypothesis is that isoform-specific disruptions by point mutations and small intragenic deletions within the *SHANK3* gene contribute to the clinical heterogeneity in humans and variable phenotypes seen in mice.

As a first step to test this hypothesis, we conducted a series of experiments to systematically characterize the extent and regulation of isoform-specific expression of *Shank3* in mice because of the ready availability of brain tissues and amenability of this model species to experimental manipulation. We discovered that *Shank3* undergoes extensive alternative splicing in the exons encoding for conserved protein domains. We report, for the first time, that the expression and alternative splicing of *Shank3* isoforms are brain-region and developmentally specific, activity dependent, and involve epigenetic regulation. We also found that different Shank3 isoforms displayed different subcellular distribution and differential effects on dendritic spine morphology, suggesting a different function for each isoform. We propose that isoform diversity of *Shank3* is one of the explanations for the phenotypic diversity in humans and mice carrying various *Shank3* defects.

## Methods

### Animals

All experiments in animals were conducted with approved protocols by the Institutional Animal Care and Use Committee at Duke University.

### Primary neuron culture and drug treatments

The methods for primary hippocampal and cortical neuron cultures were described previously [18]. Briefly, hippocampal and cortical tissues were dissected from newborn C57BL/6J pups between postnatal day 0 and day 1, and digested with trypsin. Tissues were pelleted by brief centrifugation and then dissociated in Neurobasal/B27 medium. Cells were plated into 60 mm dishes coated with 0.1 mg/mL poly-D-lysine at a density of 4 × 10^6^ cells/dish. Cells were treated on 8–10 days *in vitro* with either 30 mM KCl or 5 μM trichostatin A (TSA) for 16 hours. Astrocyte monolayers were derived from the hippocampus of postnatal day 7 C57BL/6J mice as described [27]. Total RNA and protein were prepared for quantitative PCR or western blot analysis.

### DNA constructs and transfection

Mouse brain cDNAs were prepared from cerebral cortex of 8-week-old mice. Specific primers for *Shank3* isoforms (*3a*, *3b*, *3c*, and *3e*) were employed to amplify PCR products containing the complete open reading frame of each isoform, and the PCR products were cloned into EGFP-C1 vectors using In-Fusion Cloning Kits (Clontech, CA, USA). COS-7 cells growing on coverslips in 6-well plates were transfected with 2 μg of different *Shank3* constructs using FuGENE HD transfection reagent (Promega, WI, USA) according to the manufacturer’s technical manual. The cells were fixed by 4% paraformaldehyde 36 hours post-transfection. Dissociated hippocampal neurons were grown on poly-D-lysine-coated coverslips. After 7 days *in vitro*, neurons were transfected with *Shank3* constructs using Lipofectamine 2000 transfection reagent (Invitrogen, CA, USA). Briefly, on the day of transfection, half of the medium was removed from each well and kept at 4°C. DNA and Lipofectamine 2000 were mixed in serum-free neurobasal medium with a ratio 1:3 (μg:μL). The mixture was added into each well and incubated for 6 hours before replacement with the previously saved conditioned medium. The cells were fixed by 4% paraformaldehyde after 14 days *in vitro*. To analyze the spine morphology, neurons were co-transfected with *Shank3* constructs and a tdTomato plasmid.

### Immunochemistry and morphology analysis of dendritic spines

Fixed neurons were permeabilized with 0.2% triton-X 100 in 1× phosphate buffered saline (PBS), blocked with 2% bovine serum albumin, and co-stained with rabbit anti-GFP (Invitrogen) and mouse anti-PSD-95 (UC Davis/NIH NeuroMab Facility) antibodies and corresponding secondary antibodies conjugated with Alexa 488 or Alexa 568. Confocal images were obtained using a 63× objective (Zeiss LSM 510 inverted) with sequential acquisition settings of 1024 × 1024 pixels. Each image was a z-series projection of 3–4 images at 0.5-μm depth intervals and averaged four times. Morphometric analysis and quantification of PSD-95 and dendritic spines were performed using Image J software (NIH, Bethesda, MD, USA) by an experimenter who was blinded to experiment conditions.

### RNA isolation and RT-PCR expression analysis

Tissues from different brain regions were dissected from coronal sections of brain slices cut by a Leica VT 1000p microtome (Leica, IL, USA). Total RNA was isolated using the TRIzol method (Life Technologies, CA, USA). Reverse transcription was performed with SuperScript® III first-strand synthesis system (Invitrogen). Real-time quantitative RT-PCR (q-PCR) was carried out using a LightCycler 480 Instrument (Roche Diagnostics, Mannheim, Germany) and QuantiFast SYBR green PCR kit (Qiagen, CA, USA), following the manufacturer’s recommendations. The primers used in this study are listed in Additional file 1: Table S1. AccuPrime GC-Rich DNA Polymerase (Invitrogen) was employed to amplify the full-length GC-rich sequence of exons 10–12 of *Shank3*. The sequences of newly identified splice variants were annotated and deposited in Genbank. Quantification of the bands of splicing variants were carried out using Image J [28].

### Western blot

Western blot was performed as previously described [29]. Briefly, brain tissues were homogenized and sonicated in modified RIPA buffer (1× PBS, 1% Triton X-100, 0.1% SDS, 2 mM EDTA, and protease inhibitors); 25 μg of proteins were resolved by PAGE and transferred onto polyvinylidene difluoride (PVDF) membranes. The PVDF membranes were blocked with 5% milk and incubated with Shank3 (1:5,000 in 5% non-fat milk) antibody (sc-30193, Santa Cruz, Dallas, TX, USA) at 4°C overnight. Following incubation with horseradish peroxidase-conjugated secondary antibody, the membranes were incubated with a Pierce chemiluminescent substrate (Rockford, IL, USA) and exposed to X-ray film.

## Results

### Multiple protein isoforms produced by intragenic promoters in mouse brain

**Figure 1:**
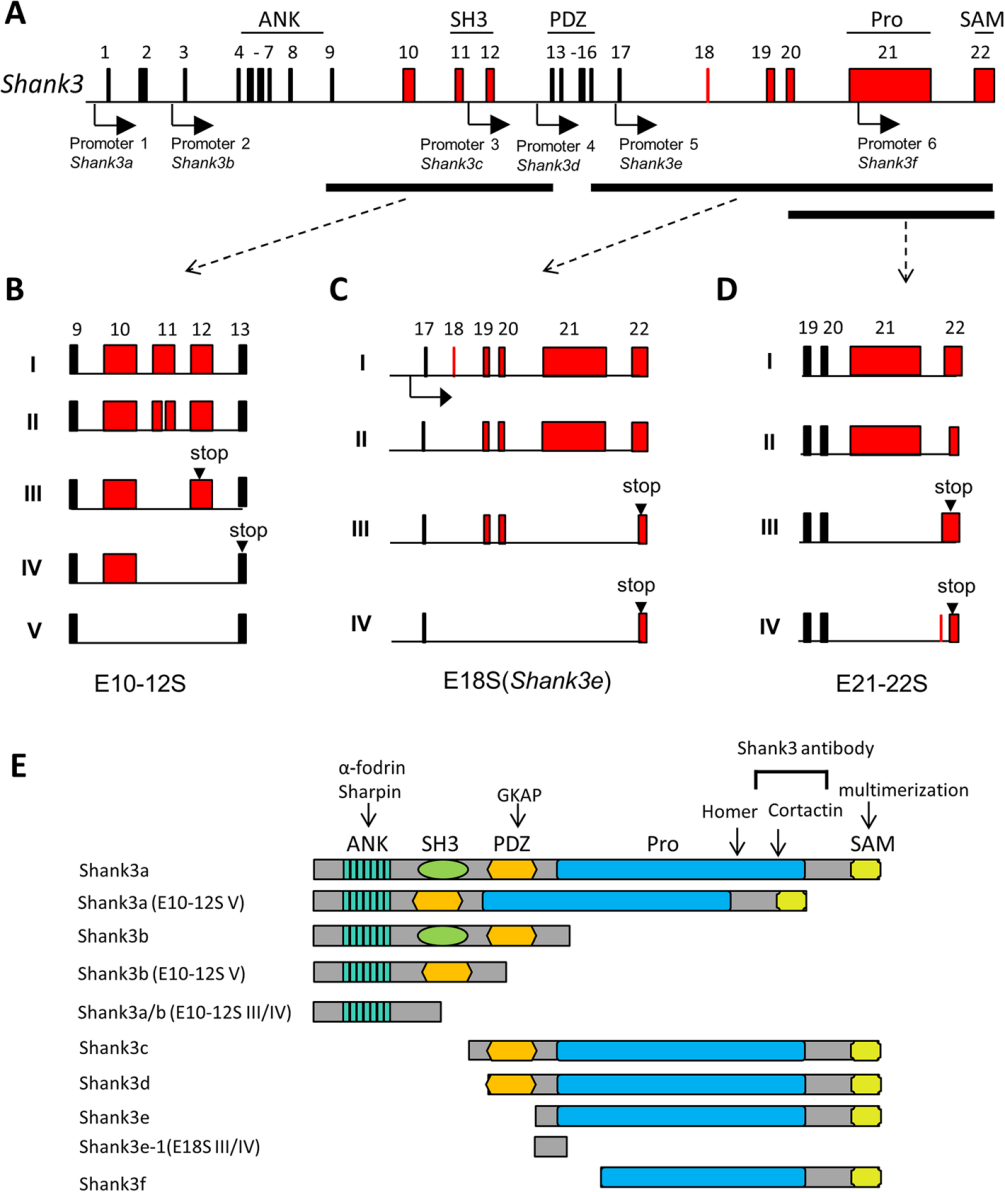
**Diverse *Shank3 *isoforms result from intragenic promoters and alternative splicing. (A)** Multiple promoters of the *Shank3* gene. Mouse *Shank3* has 22 coding exons as illustrated. The exon-intron structure was deduced by cDNA AB231013 in mm10 mouse genome assembly ( http://www.genome.ucsc.edu). The alternatively spliced exons are highlighted in red. The positions of intragenic promoters are shown as black arrows. Protein domains encoded by exons are labeled above the gene structure. **(B–D)** Extensive alternative splicing of *Shank3* coding exons. **(B)** *Shank3* exons 10–12 splice variants (E10–12S I to V). The pattern was deduced from the sequences of PCR products in Figure S2A (Additional file 3). I: no splicing. II: exon 11 partially spliced out. III: exon 11 spliced out. IV: exons 11 to 12 spliced out. V: exons 10 to 12 spliced out. **(C)** *Shank3e* splice variants (E18S I to V). The pattern was deduced from the sequences of PCR products in Figure S2C (Additional file 3). I: no splicing. II: exon 18 spliced out. III: exons 18, 21, and 22 (partial) spliced out. IV: exons 18 to 21 and 22 (partial) spliced out. **(D)** *Shank3* exon 21 and exon 22 splice variants (E21–22S I to IV). The pattern was deduced from the sequences of PCR products in Figure S2E (Additional file 3). I: no splicing. II: exon 22 (partial) spliced out. III: exon 21 spliced out. IV: exon 21 and exon 22 (partial) spliced out. **(E)** The predicted Shank3 protein isoforms were deduced from the combination of intragenic promoters and alternative splicing of mRNAs described above and *in silico* analysis. The binding positions of several well-known PSD proteins are shown by arrows. The epitope position for the Shank3 antibody used in this study is also indicated.

The mouse *Shank3* gene has 22 exons that encode a synaptic scaffolding protein with five functional domains (Figure 1A). We have previously demonstrated that the *Shank3* gene contains at least five intragenic promoters in humans and rodents which produce promoter-specific isoforms of Shank3 with various combinations of different functional domains [8,18,25] as summarized in Figure 1E. The presence of multiple intragenic promoters is supported by CHIP sequencing data from the ENCODE project (Additional file 2: Figure S1B, http://genomebrowser.wustl.edu). The analysis of ENCODE data also suggests that these intragenic promoters are brain specific, which was confirmed by western blot analysis of Shank3 protein among different tissues (Additional file 2: Figure S1C). The presence of intragenic promoters *in vivo* is also supported by the expression analysis in a list of *Shank3* isoform-specific mutant mice. In these mutant mice, different portions of *Shank3* exons were deleted, resulting in disruption of select sets of Shank3 proteins and mRNAs [16-20]. In support of these findings, *Shank3* complete knockout mice with deletion of exons 4–22 showed absence of all known promoter-specific isoforms (data not shown).

### Extensive alternative splicing of *Shank3* mRNAs confers further complexity to *Shank3* isoforms

Alternative splicing of *Shank3* has been suggested [12,18] but has not been fully characterized. We conducted RT-PCR with primer combinations that cover all exons of *Shank3* (Additional file 2: Figure S1A) using total RNA from the cerebral cortex of 8-week-old mice. We discovered that the coding exons 10–12, exon 18, exon 21, and exon 22 of *Shank3* displayed extensive alternative splicing (Additional file 3: Figure S2). Interestingly, the alternatively spliced exons were concentrated in the conserved SH3, proline-rich, and SAM domains of Shank3 and resulted in protein species with different combinations of the five functional domains. The alterative splicing of exons 10–12 resulted in five different splice variants (E10–12S I to V, Figure 1B). E10–12S I represented the full length of this portion of mRNA without alternative splicing. In E10–12S II, a cryptic splicing of 57 nucleotides occurred in exon 11 without a shift of the open reading frame (ORF) of *Shank3* mRNA. However, splice variants of E10–12S III and IV resulted in truncated isoforms only containing the ANK domain due to a frame shift of the *Shank3* ORF (Figure 1E). Interestingly, skipping of exons 10 to 12 in E10–12S V was predicted to produce a Shank3 isoform without the SH3 domain but presumably retaining the other four protein domains (Figure 1E). Alternative splicing of exons 18–22 was examined by primers specific to promoter 5 for *Shank3e* (Additional file 2: Figure S1A). Four splice variants (E18S I to IV, Figure 1C) were identified. Exon 18 was spliced out in E18S II to IV. The splicing of exon 18 appeared to be in concert with splicing of exons 21/22 or exons 19–22 in *Shank3* E18S III and IV, respectively. The frame shift of the *Shank3* ORF in E18S III and IV variants is predicted to result in premature stop codons in exon 22 and produced short Shank3e isoforms (Shank3e-1) lacking the proline-rich region and SAM domain (Figure 1E). With a different set of primers, alternative splicing of exons 21 and 22 was examined, and four different variants were observed (E21–22S I to IV, Figure 1D). While the E21–22S I and II variants did not change the ORF of *Shank3*, the E21–22S III and IV variants were predicted to produce C-terminal truncated Shank3 isoforms lacking the SAM domain due to premature stop codons in exon 22. In summary, these results indicate that *Shank3* undergoes extensive alternative splicing, which results in extreme diversity of *Shank3* isoforms at mRNA and possibly at protein levels.

### Region- and development-specific expression of *Shank3* isoforms in mouse brain

**Figure 2:**
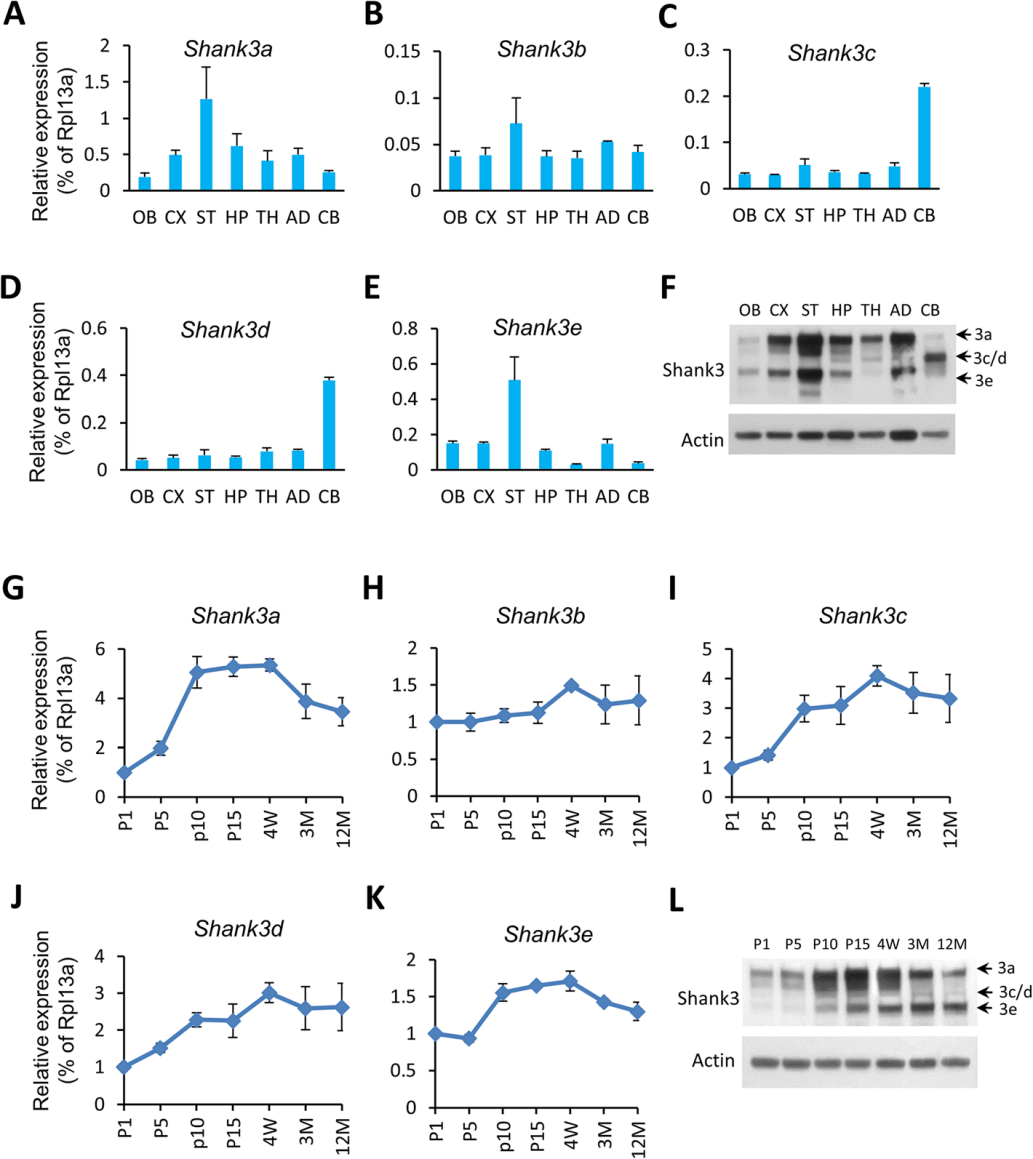
**Differential expression of Shank3 isoforms across brain regions and development. (A–E)** q-PCR of *Shank3* major isoforms in different brain regions. **(G–K)** q-PCR of *Shank3* isoforms in brain across development. The crossing point (CP) values of each isoform were normalized to that of a housekeeping gene, *Rpl13a*. For brain regions: OB, olfactory bulb; CX, cortex; ST, striatum; HP, hippocampus; TH, thalamus; AD, amygdala; CB, cerebellum. For different ages: P1, postnatal day 1; P5, postnatal day 5; P10, postnatal day 10; P15, postnatal day 15; 4 W, 4 weeks; 3 M, 3 months; 12 M, 12 months. **(F** and **L)** Western blot analysis using an antibody produced from an epitope at the C-terminus of SHANK3 protein (amino acids 1431 to 1590) for different mouse brain regions **(F)** or at different ages **(L)***.* The predicted bands for Shank3a (185KD), Shank3c/d (128KD/127KD), and Shank3e (118KD) are indicated by arrows. All of these bands migrate a little higher than prediction, which may be caused by post-translational modifications of Shank3 proteins. Shank3b was not detectable as the epitope of the antibody was spliced out in this isoform. All data are shown as mean ± SEM.

Regional and developmental expression of *Shank3* has been previously examined by RNA *in situ* hybridization [17,30]. However, the probes used in these studies could not distinguish between different *Shank3* isoforms. To examine the isoform-specific expression of *Shank3* in different brain regions, we performed isoform-specific q-PCR using 8-week-old mice (Figure 2A–E). *Shank3a* and *Shank3e* were highly expressed in striatum but low in olfactory bulb and cerebellum. In contrast, *Shank3c* and *Shank3d* were predominantly enriched in cerebellum. *Shank3b*, an isoform without exon 21 encoding the homer binding site, is expressed at low levels throughout the brain. We were not able to design primers to examine mRNA from *Shank3f* because this promoter is embedded within exon 21. Western blot analysis using an antibody against the C-terminus of Shank3 revealed an expression pattern that is highly correlated with the pattern of q-PCR (Figure 2F). Specifically, Shank3a and Shank3e were predominant in striatum and Shank3c/d was exclusively enriched in the cerebellum. Parenthetically, the diversity of Shank3 protein products may be under-represented because some truncated Shank3 protein isoforms are not detectable due to the location of the epitope of the Shank3 antibody used. Developmental expression of *Shank3* was also examined by q-PCR using whole brains from mice of different ages (Figure 2G–K), and all isoforms showed an increase from postnatal day 1 to 4 weeks. The peak of expression was between 2 and 4 weeks, correlating with the time frame for synaptogenesis. *Shank3a* and *Shank3e* displayed a gradual decrease after 4 weeks and reduced to 50% of the peak level at 12 months of age. In contrast, the expression of *Shank3c* and *Shank3d* was relatively stable throughout adulthood. Western blot revealed a similar pattern of Shank3 protein isoforms at different postnatal ages in mouse brain (Figure 2L). The temporally and spatially specific expression of *Shank3* isoforms suggests different functions for different isoforms.

### Region-, development-, and cell-type-specific alternative splicing of *Shank3* isoforms in mouse brain

**Figure 3:**
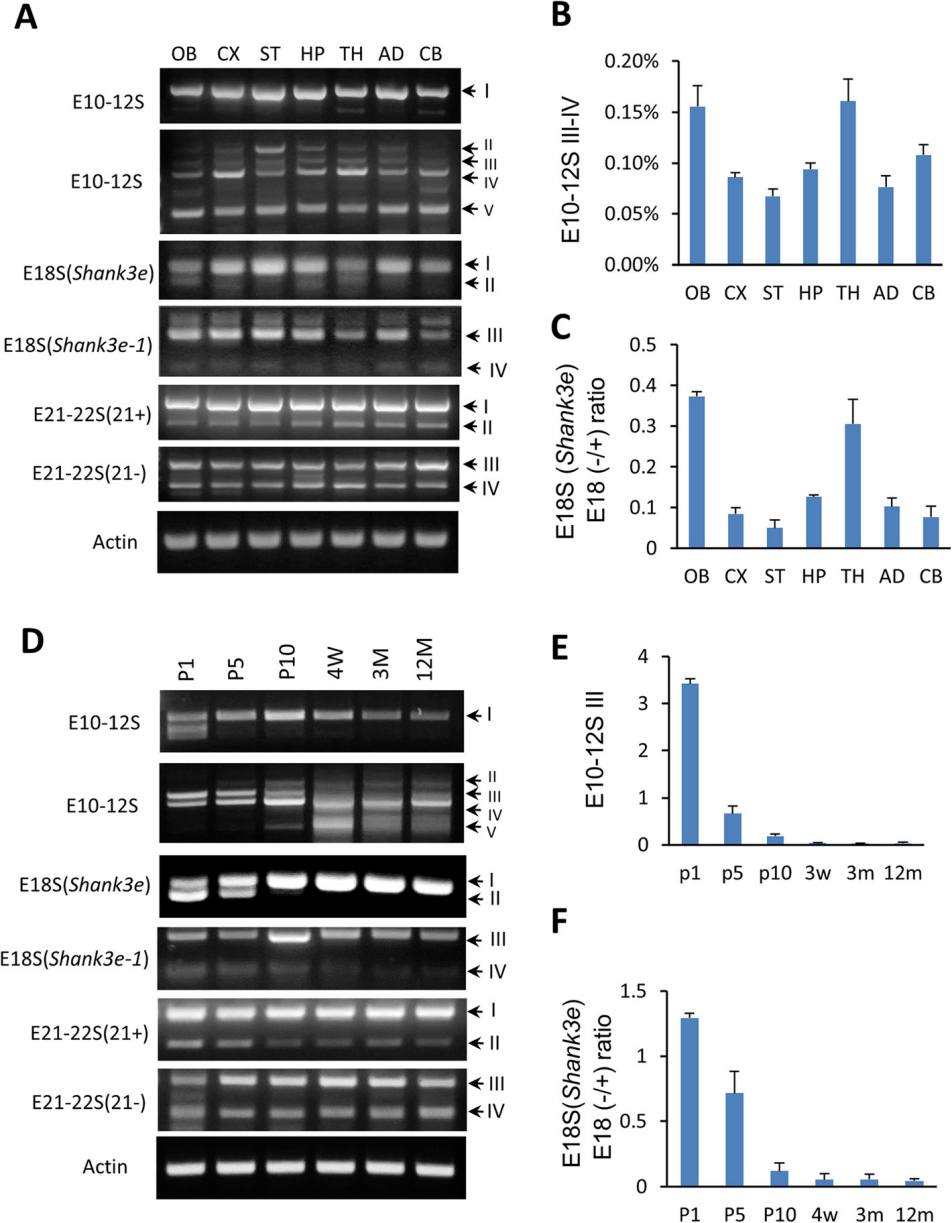
**Alternative splicing of *Shank3 *in different brain regions and across development. (A)** Splicing of *Shank3* coding exons in different brain regions. **(B)** Quantification for splicing of E10–12S III to IV in **(A)**. The values of the spliced bands were normalized to E10–12S I without splicing. **(C)** Quantification for splicing of exon 18 in **(A)***.* Values were shown as the ratio of exon18 (-) to exon18 (+). **(D)** Splicing of *Shank3* coding exons during brain development. **(E)** Quantification for splicing of E10–12S III in **(D)**, values of the spliced products were normalized to that of E10–12S I without splicing. **(F)** Quantification for splicing of exon 18 of *Shank3e* in **(D)***.* All data are shown as mean ± SEM. The abbreviations for different brain regions and ages are the same as in Figure 2.

We next investigated whether alternative splicing of *Shank3* isoforms also displays a temporally and spatially specific pattern in mouse brains. In the adult brain, the splicing of E10–12S III to IV showed a higher rate in the olfactory bulb and thalamus as compared to other regions (Figure 3A, B). The splicing of E18S II variants displayed a similar pattern (Figure 3A, C). During development, the splice variants E10–12S III and E18S II were prominent at postnatal day 1, then rapidly decreased at postnatal day 5 and only expressed at marginal levels after postnatal day 10 (Figure 3D, E). The ratio of exon 18– (E18S II) versus exon 18+ (E18S I) was inverted from postnatal day 1 to postnatal day 5 (Figure 3F). The expression of E10–12S V significantly increased toward 4 weeks and decreased in the adult brain (Figure 3D). The splice variants of E18S III and IV were also differentially regulated during development. In addition, alternative splicing of *Shank3* isoforms also displayed a cell-type-specific pattern (Additional file 4: Figure S3). Interestingly, alternative splicing of exon 18 was mutually exclusive between cell types, with exon 18 inclusion (E18S I) in neurons and exon18 exclusion (E18S II) in astrocytes. Similarly, the E18S III variant was exclusively observed in neurons while E18S IV was only found in astrocytes. Taken together, these results showed that alternative splicing of *Shank3* is region-, development-, and cell-type-specific.

### Activity dependent expression and alterative splicing of *Shank3*

**Figure 4:**
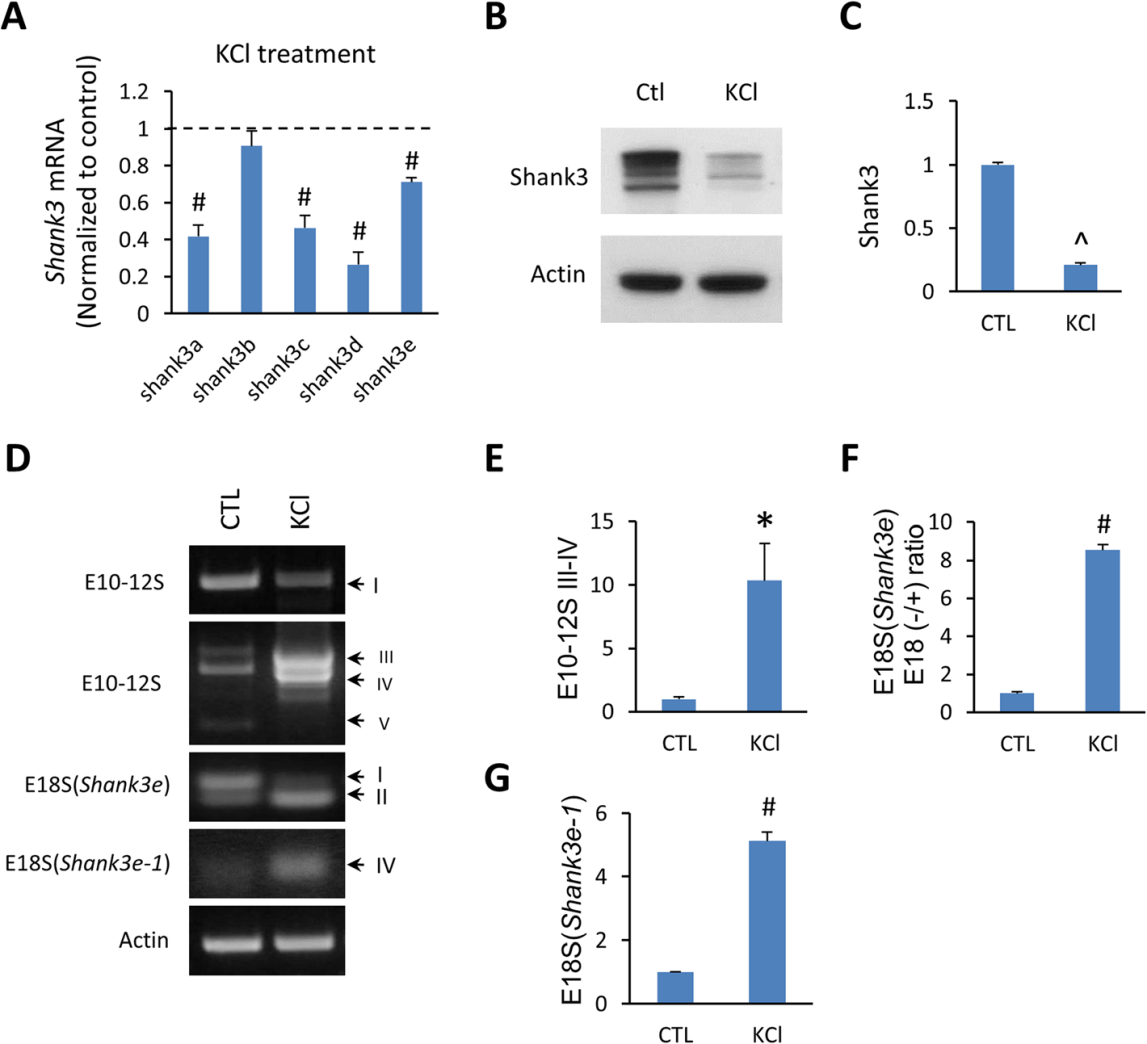
**Neuronal activity-dependent isoform-specific expression and alternative splicing of *Shank3*. (A)** q-PCR analysis of *Shank3* isoforms in cultured mouse cortical neurons after treatment with 30 mM KCl for 18 hours*.* The expression of *Shank3a*, *3c*, *3d*, and *3e* was significantly reduced while no change was observed for *Shank3b*. **(B)** Western blot showed reduced Shank3 proteins after KCl treatment. **(C)** Quantification for Shank3 proteins in **(B)***.***(D)** Splicing of *Shank3* isoforms after KCl treatment. Alternative splicing for E10-12S III-IV **(E)**, E18S II of *Shank3e***(F)**, and E18S IV of *Shank3e-1***(G)** were increased after KCl treatment. All data are shown as mean ± SEM. *P <0.05, #*P* <0.002, ^*P* <0.001, compared to control group, two tail *t*-test, n = 4 each group.

The expression of many neuronal genes is regulated by neuronal activity [31,32]. To investigate if the splicing and expression of *Shank3* are also regulated by neuronal activity, we treated cultured hippocampal neurons with KCl. At the mRNA level, the expression of *Shank3a*, *3c*, *3d*, and *3e* in stimulated neurons was reduced to 40% to 70% of controls while the expression of *Shank3b* was not affected (Figure 4A). Similarly, Shank3 protein isoforms were markedly reduced after KCl treatment (~20% of controls) (Figure 4B, C). The reduction of Shank3 protein after KCl treatment was more pronounced than that of *Shank3* mRNAs. The reason underlying the difference between the mRNAs and proteins was not immediately clear. Possibilities involving post-translational regulation of Shank3 protein may be considered. For example, ubiquitin/proteasome-mediated protein degradation of Shank family proteins following neuronal activity has been observed [31]. Alternative splicing of *Shank3* was also affected by KCl treatment (Figure 4D). A significant increase in the splice variants E10–12S III to IV, E18S II, and E18S IV was observed after KCl treatment (Figure 4E–G). In contrast, the splice variants E21–22S were not affected (data not shown). These results indicate that the expression and alternative splicing of *Shank3* isoforms are regulated by neuronal activity.

### Expression and alternative splicing of *Shank3* are regulated by a histone deacetylase (HDAC) inhibitor

**Figure 5:**
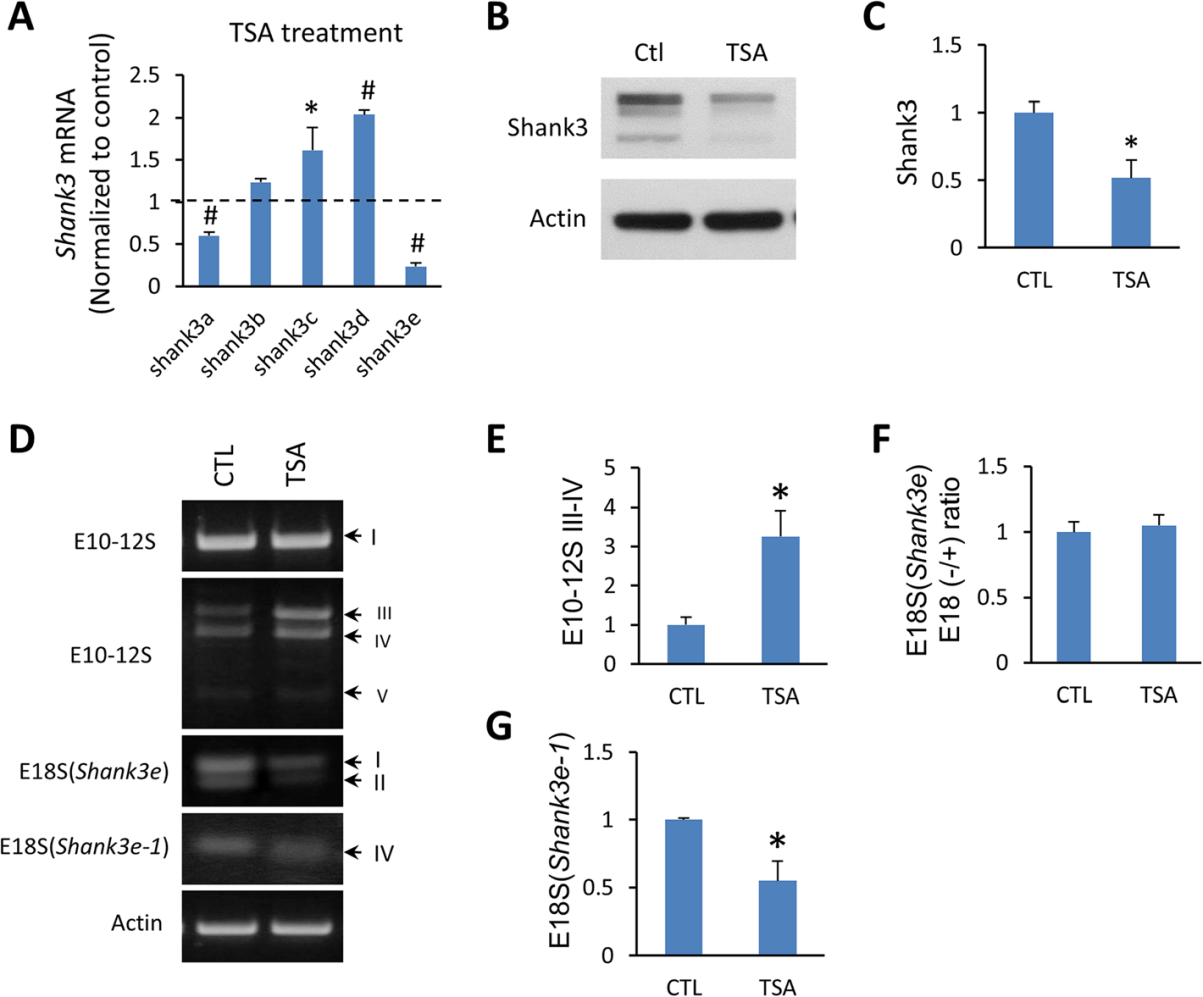
**Differential isoform-specific expression and alternative splicing of *Shank3 *induced by a histone deacetylase (HDAC) inhibitor. (A)** q-PCR analysis of *Shank3* isoforms in cultured mouse cortical neurons after treatment with 5 μM trichostatin A (TSA), a potent HDAC inhibitor, for 18 hours*.***(B)** Western blot showed reduced Shank3 proteins after TSA treatment. **(C)** Quantification for Shank3 proteins in **(B)***.***(D)** Differential changes of alternative splicing of *Shank3* after TSA treatment. **(E)** Alternative splicing for *Shank3* E10–12S III to IV was increased. **(F**) Splicing for E18S II of *Shank3e* was not changed. **(G)** Splicing for E18S IV of *Shank3e-1* was decreased. All data are shown as mean ± SEM. * *P* <0.05, # *P* <0.002, compared to the control group, two tail *t*-test, n = 4 each group.

Epigenetic modifications, such as histone acetylation, have been shown to regulate expression and alternative splicing of neuronal genes [33,34]. To examine whether expression and alternative splicing of *Shank3* are subject to epigenetic regulation, we treated hippocampal neurons with TSA, a potent HDAC inhibitor. Interestingly, TSA treatment exerted differential effects on the isoform-specific expression of *Shank3*. At the mRNA level, *Shank3a* and *Shank3e* were significantly reduced while *Shank3c* and *Shank3d* were markedly increased (Figure 5A). The expression of *Shank3b* was not affected. Reduced *Shank3* expression was also supported by the overall reduction of Shank3 proteins (Figure 5B, C). However, isoform-specific changes were not apparent by western blot analysis. The discrepancy between the mRNA and protein expression is not immediately clear. The possibility of antibody specificity and sensitivity may be considered. Alternatively, the involvement of protein modification for different protein isoforms related to HDACs may also be a factor. TSA treatment also induced an increase in E10–12S III to IV and a reduction of E18S IV (Figure 5D, E, G). Splicing of E18S II was not changed despite a marked decrease of *Shank3e* mRNA (Figure 5D, F). Alternative splicing of *Shank3* E21–22 was not affected by TSA treatment (data not shown). Taken together, the expression and splicing of *Shank3* isoforms were differentially regulated by an HDAC inhibitor, suggesting the involvement of epigenetic modifications in regulating *Shank3* expression, but the exact mechanisms are not immediately clear and further investigation is warranted.

### Differential subcellular localization of Shank3 isoforms and their effects on dendritic spines

**Figure 6:**
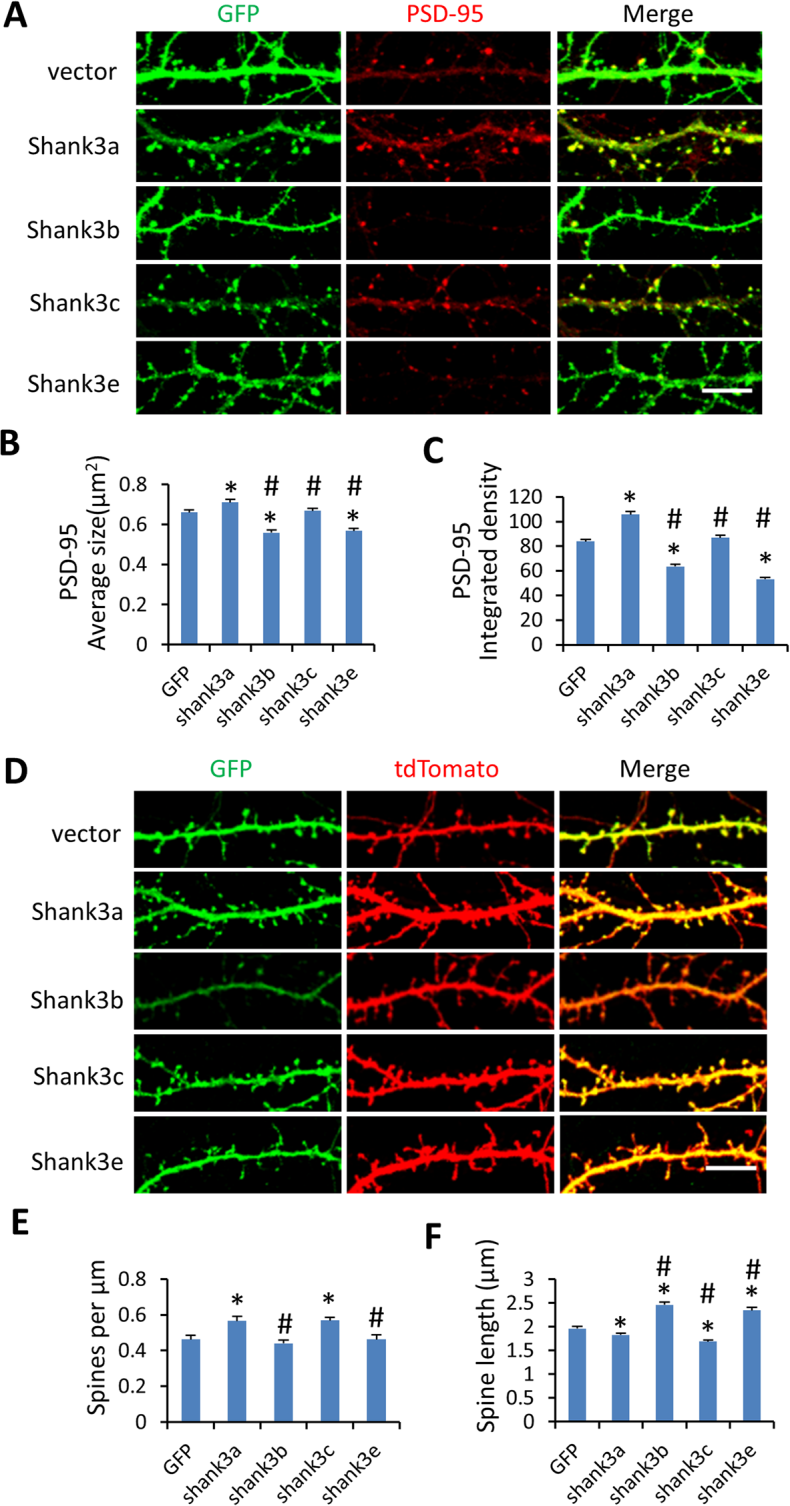
**Differential contributions of Shank3 isoforms to excitatory synapses. (A)** Co-localization of Shank3 isoforms with PSD-95. Shank3 isoforms tagged with GFP (green) were expressed in primary hippocampal neurons and co-stained with an excitatory post-synaptic marker PSD-95 (red). Shank3 isoforms displayed differential impacts on the size **(B)** and density **(C)** of PSD-95 puncta. **(D)** Impacts of Shank3 isoforms on dendritic spine morphology. EGFP-Shank3 isoforms (green) were co-transfected with tdTomato (red) in hippocampal neurons. Shank3 isoforms displayed differential effects on spine density **(E)** and spine length **(F)** of hippocampal neurons. All data are shown as mean ± SEM. *, *P* <0.01, compared to GFP; #, *P* <0.01, compared to Shank3a, two tail *t*-test. Scale bar: 10 μm.

To analyze the subcellular distribution of Shank3 isoforms and their roles in regulation of dendritic spines, we cloned cDNAs of major *Shank3* isoforms into EGFP-C1 vectors. First, we overexpressed these constructs in COS-7 cells (Additional file 5: Figure S4A). As a control, GFP showed a diffuse pattern without cluster formation, while Shank3a, c, and e, all containing the proline-rich region and SAM domain, formed clusters in the cytoplasm. Overexpression of *Shank3* exon 22 only encoding the SAM domain displayed a similar pattern, indicating the clusters were mediated by the SAM domain through homo-/hetero-polymerization [1]. Interestingly, Shank3b was localized in the nucleus as it overlapped with DAPI, a nucleus-staining marker. This suggests that the lack of proline-rich and SAM domains may be responsible for its nuclear targeting. The nuclear localization of Shank3b was also verified when it was expressed in cultured hippocampal neurons (Additional file 5: Figure S4B). To determine whether any Shank3 isoform is present in inhibitory synapses or not, we examined the overlap of Shank3 isoforms with gephyrin, an inhibitory post-synaptic marker. None of the Shank3 isoforms examined localized in inhibitory synapses of cultured neurons (Additional file 6: Figure S5). However, it remains to be investigated whether the same is true *in vivo* in mouse brain due to the technical caveats of cultured cells. The co-localization of PSD-95, a marker of excitatory post-synapses, with Shank3a and Shank3c was observed in dissociated hippocampal neurons but was not apparent with Shank3b and Shank3e (Figure 6A). The size and the density of PSD-95 puncta were increased in Shank3a transfected neurons, indicating an increase in excitatory synapses. On the contrary, overexpression of Shank3b and Shank3e resulted in a reduction of the size and density of PSD-95 (Figure 6B, C). We further examined the contribution of different Shank3 isoforms in dendritic spine development by co-transfection of Shank3 isoforms and tdTomato in hippocampal neurons (Figure 6D). Shank3a and Shank3c caused an increase in spine density and a slight decrease in spine length compared to the EGFP control. Shank3b and Shank3e did not cause significant changes in the spine density (*P* = 0.47 and 0.97, respectively) but resulted in a marked increase in spine length (Figure 6E, F), indicating the formation of more immature synapses. These results indicate that Shank3 isoforms have different subcellular localization and differential effects on dendritic spine development, further suggesting that they play different roles in the brain.

## Discussion

Using various molecular genetics approaches, we have described an unusually complex transcriptional profile for *Shank3*, a strong human autism causative gene, in the mouse brain. We have delineated transcript structures for major *Shank3* isoforms resulting from the combination of multiple intragenic promoters and alternative splicing. Furthermore, we showed, for the first time, that the isoform-specific expression of *Shank3* is temporally and spatially specific and regulated by neural activity. We showed that the isoform-specific expression of *Shank3* in cortical neurons involves an epigenetic regulation suggested in previous reports [23,35]. Further, this is the first report indicating that a selective Shank3 isoform is strictly localized in neuronal nuclei which implies a novel function other than as a scaffolding protein at the PSD. Our results support the hypothesis that different Shank3 isoforms have distinct functions at synapses. These findings will promote our knowledge of the molecular diversity in the brain and help us to understand the phenotypic heterogeneity caused by various *SHANK3* defects in humans and mice.

### The implication of molecular complexity of *Shank3* to the functional diversity of synapses

Our results reveal that *Shank3* displays more isoforms in the brain than it does in peripheral tissues and suggest that each Shank3 isoform has a unique function, which allows us to propose that the complexity of *Shank3* contributes to the functional diversity of synapses. First, the major isoforms of Shank3 generated from different promoters contain distinct combinations of the five conserved domains, which provide the functional diversity for Shank3 isoforms. The full-length Shank3a contains all five domains which would have the capacity to interact with all possible interacting proteins. Other isoforms with different combinations of functional domains would then interact with a different subset of synaptic proteins. For instance, the N-terminal truncated Shank3e only contains the proline-rich region and SAM domain and would be able to couple with homer-mGluRs complexes but not GKAP-PSD95-NMDARs complexes [4,5]. In contrast, the C-terminal truncated Shank3b would be able to interact with NMDARs but not mGluRs. By competing with Shank3a, one of the functions of Shank3b and Shank3e might be the regulation of the cross-talk between NMDARs and mGluRs, which results in the fine tuning of synaptic transmission. Second, alternative splicing contributes to further complexity of Shank3 isoforms and functional diversity. Alterative splicing of *Shank3* is predicted to have an impact on the function of *Shank3* since it is heavily concentrated in the exons encoding functional protein domains. The splice variants of E10–12S III and IV generate truncated Shank3 isoforms which only contain the ANK domain known to interact with cytoskeleton proteins such as alpha-fodrin and sharpin [30,36]. Conceptually, these isoforms will compete with the binding of full-length Shank3 to alpha-fodrin and sharpin, thereby modulating the rearrangement of the PSD structure. It is interesting that these isoforms are highly expressed at early stages of brain development and concentrated in the olfactory bulb of adult brain. The splicing variant E18S II showed similar region- and development-specific splicing patterns to that of E10–12S, implying that they may coordinate with each other for some functions. Exon 18 inclusion may be considered a neuronal marker at the mRNA level, as it occurs specifically in neurons, but not in astrocytes or peripheral tissues [12]. Exon 18 contains 24 nucleotides that encode 8 amino acids containing an arginine stretch (RRRK) residues that is similar to the "RXR" motif. It has been shown that the RXR motif serves as an endoplasmic reticulum (ER) retention signal in the NMDA receptor subunit NR1 [37]. Whether or not this motif regulates the sorting and trafficking of Shank3 *in vivo* is an interesting question for further investigation. Third, the functional diversity of Shank3 isoforms is supported by the finding that Shank3 isoforms display different subcellular localization and differential effects on spine morphology. For example, the full length Shank3a increased the density of dendritic spines and PDS-95 puncta, but Shank3b and Shank3e have an opposite impact on dendritic spine development. The nuclear targeting of Shank3b in heterologous cells and neurons implicates that this isoform probably possesses a function other than as a scaffolding protein at the PSD as described to date. In addition, the temporally- and regionally-specific expression of *Shank3* isoforms also supports the concept that different isoforms have different functions. Altogether, our data strongly support a notion that different Shank3 isoforms contribute differentially to synaptic function. Follow-up studies are warranted to determine the distinct role for each isoform in synaptic development and function.

### The complexity of *Shank3* and the specification of synapses

Although the phenomena of alternative splicing and multiple promoters are commonly described in both neuronal and non-neuronal genes, the degree of the complexity described for *Shank3* is somewhat unusual. A similar level or even more complexity has been described for genes such as Neurexin family proteins and Brain Derived Neurotrophic Factor [38,39]. The exact number of *Shank3* mRNAs and protein isoforms is not known. Whether all mRNA isoforms are translated into proteins cannot be easily determined. Given the tissue-/cell-type and developmental-stage specific promoter usage and alternative splicing, the number of Shank3 isoforms could be very substantial and possibly reach to more than a hundred. The interesting question is whether there is a biological purpose underlying such complexity as has been observed for *Shank3*. Humans have billions of neurons and a trillion synapses but a relatively small number of genes in a genome (approximately 20,000). We have a limited understanding of what contributes to the diversity of synapses, and the current methods to classify different types of synapses are extremely simplified. From revealing the complexity of Shank3 and other synaptic proteins, one plausible hypothesis is that the myriad isoforms of Shank3 and other synaptic proteins are destined to contribute to the diversity or specification of types of synapses. One could speculate that the different isoforms of Shank3 may localize differentially in the nano-structure of the PSD, such as central versus peripheral sites in the PSD of the same synapse, or different isoforms of Shank3 are at different excitatory synapses in a given pyramidal neuron. In addition, some protein isoforms may be present only when the neurons are stimulated with certain types of neural activity that lead to local protein translation for specific *Shank3* mRNA isoforms. To examine these possibilities, high resolution cellular imaging at single synapses is required and isoform-specific antibodies will be helpful to assist these analyses.

### The complex *Shank3* transcriptional regulation and phenotypic heterogeneity of *Shank3* mutant mice and *SHANK3* causing ASD

Considerable clinical heterogeneity in ASD has been well documented [40-42], although the cause underlying this heterogeneity remains largely unknown. Molecular heterogeneity due to the number of genes has been hypothesized to be one of the logical explanations [43,44], and has been supported by the findings of whole exome or whole genome sequencing of ASD cases [45-48]. However, in the case of *SHANK3* causing ASD, considerable heterogeneity is also observed among the cases with different mutations within the *SHANK3* gene [8]. We have previously shown that human *SHANK3* also displayed a similar pattern of promoter usage and alternative splicing [25]. With the knowledge of the complexity of *Shank3* transcriptional regulation reported here, the clinical heterogeneity of *SHANK3* causing ASD could be predicted to result from the disruption of different sets of *SHANK3* isoforms due to the location of the mutation within the coding exons of *SHANK3*. This prediction has been supported by *Shank3*-isoform mutant mouse models, in which phenotypic diversity was demonstrated among mutant mice with disruption of different isoforms [16-20]. A striking example is that disruption of *Shank3a* to *Shank3c* results in skin lesion/increased self-grooming phenotype, but this phenotype is absent in *Shank3a* to *Shank3b* deficient mice, implicating the unique role of *Shank3c* in the development of self-induced skin lesions. Although genetic background and environmental factors may also contribute to the phenotypic heterogeneity in *SHANK3* causing ASD and *Shank3* mutant mice, our analysis of Shank3 isoforms provides a framework at the molecular level to understand the question of phenotypic heterogeneity. A head-to-head comparison between different *Shank3* mutant mice in the same genetic background and *in vivo* analysis of the function of each Shank3 isoform will yield insights into the mechanism underlying phenotypic heterogeneity in *SHANK3* causing ASD.

## Conclusions

In summary, we showed a complex transcriptional regulation of *Shank3* in mouse brain that resulted in diverse Shank3 isoforms. The regional, developmental, activity-dependent, and epigenetic modulation of isoform-specific expression and alternative splicing of *Shank3* suggest a different function for each Shank3 isoform. It is predicted that the *SHANK3* defects in reported ASD patients and *Shank3* mutant mice are isoform-specific. Our study then provides a molecular framework to dissect clinical heterogeneity of *SHANK3* causing human disorders and provide insight to better understand the molecular basis underlying the clinical heterogeneity of ASD in general. In addition, these findings are critically important to interpret the difference between different lines of *Shank3* mutant mice and formulate the plan to further analyze these mutant mice to elucidate the contribution of *Shank3* to the pathophysiology of ASD.

## Abbreviations

ANK: Ankyrin repeat; ASD: Autism spectrum disorder; HDAC: Histone deacetylase; mGluR: Metabotropic glutamate receptor; NMDAR: N-methyl-D-aspartate receptor; ORF: Open reading frame; PSD: Postsynaptic density; PDZ: PSD-95/Discs large/ZO-1; q-PCR: Quantitative real-time polymerase chain reaction; SAM: Sterile alpha motif; SH3: Src homology 3; TSA: Trichostatin A.

## Competing interests

The authors declare that they have no competing interests.

## Authors’ contributions

XW: Conception and design, data collection and analysis, manuscript writing, critical revision, and final approval of the manuscript. QX: Data collection and analysis, manuscript writing, and final approval of the manuscript. ALB: Data collection, manuscript writing and editing, critical revision, and final approval of the manuscript. YL: Data collection, manuscript writing, and final approval of the manuscript. YHJ: Conception and design, financial support, manuscript writing, and final approval of the manuscript. All authors read and approved the final manuscript.

## Supplementary Material

Additional file 1: Table S1 List of *Shank3* primers used in this study. Click here for file

Additional file 2: Figure S1 Brain-specific expression of *Shank3* intragenic promoters. **(A)** *Shank3* gene structure. Intragenic promoters are shown as black arrows. Exons in red are alternatively spliced exons. In lower panel, positions of primers for *Shank3* isoforms and splice variants used in this study are indicated. Note that splicing forward primer (in red) for E18S is specific for promoter 5. **(B)** The presence of multiple intragenic promoters supported by *in silico* data from ENCODE project using mouse mm10 genome assembly ( http://genomebrowser.wustl.edu/). The CHIP sequence data using antibodies against RNA polymerase II (Pol II) and trimethylated lysine 4 of histone 3 (H3K4me3), two landmarks for active promoters, displayed several corresponding peaks (blue rectangular, dash line indicates a weak promoter) near *Shank3* promoters (arrows below *Shank3* gene) in brain tissues but not in heart and kidney, indicating these intragenic promoters are brain specific. **(C)** Brain specific expression of Shank3 isoforms from intragenic promoters. Left panel: Western blot analysis using a Shank3 antibody against C-terminus of SHANK3 (sc-30193, Santa Cruz, CA, USA) revealed multiple bands across different tissues. The bands with predicted size of Shank3c, 3d, and 3e are observed in cerebral cortex and cerebellum but not in heart, liver, and kidney. Shank3b is not detectable because it does not contain the C-terminal sequence of Shank3 protein. This result supports that the intragenic promoters are brain-specific. Right panel: Ponceau S staining of the PVDF membrane on the left panel to show that an equal amount of protein (30 μg) was loaded to each lane. Note there is variation of endogenous protein composition across different tissues. Click here for file

Additional file 3: Figure S2 Extensive splicing of *Shank3* mRNAs. **(A–B)** *Shank3* exons 10–12 spliced (E10–12S) variants. At least five different products of E10–12S are identified by RT-PCR **(A)** and the sequences of E10–12S variants were illustrated **(B)**. Arrows indicate the position of primers. I: no splicing. II: exon 11 partially spliced out. III: exon 11 spliced out. IV: exons 11 to 12 spliced out. V: exons 10 to 12 spliced out. Note that regular PCR reactions yield mostly spliced variants as the full-length of this sequence without alternative splicing can only be amplified in GC-rich buffer due to the extremely high GC percentage (79%) in exon 11 (see Materials and methods). **(C–D)** *Shank3e* spliced variants (E18S). Different sets of primers yielded various PCR products **(C)**: left panel with primers 1 and 2, right panel with primers 1 and 3. The forward primer 1 is specific for *Shank3e* isoforms from promoter 5. Gene structure of full-length *Shank3e* and its spliced variants are illustrated in **(D)**, with arrows showing the position for primers. I: no splicing. II: exon 18 spliced out. III: exons 18, 21, and 22 (partial) spliced out. IV: exons 18 to 21 and 22 (partial) spliced out. **E–F**, Splicing variants of *Shank3* exon 21 and exon 22 (E21–22S). The PCR products are shown in **(E)**, with left panel using primers 2 and 3, right panel using primers 1 and 3. Gene structure of exons 19–22 and its splicing variants were illustrated in **(F)**, with primers labeled as arrows. I: no splicing. II: exon 22 partially spliced out. III: exon 21 spliced out. IV: exon 21 and exon 22 (partial) spliced out. Gene bank accession numbers are shown in red for novel splicing variants identified by the current study. Accession numbers in black are splicing variants identified in the previous study by Wang et al. [18]. Click here for file

Additional file 4: Figure S3 Differential expression and alternative splicing of *Shank3* isoforms in neurons and astrocytes. mRNAs from cultured hippocampal neurons and astrocytes were analyzed by RT-PCR. All major isoforms of *Shank3* mRNAs were expressed abundantly in neurons as expected. *Shank3a*, *3c*, *3d*, *3e*, and *3e*-*1* were low but readily detectable in astrocytes. Alternative splicing of exon 18 was mutually exclusive between cell types, with exon18 inclusion (E18S I) in neurons and exon18 exclusion (E18S II) in astrocytes. Similarly, the E18S III variant was exclusively observed in neurons while E18S IV was only found in astrocytes. N, neuron. A, astrocyte. Click here for file

Additional file 5: Figure S4 Differentiated cellular distribution of Shank3 isoforms. Shank3 isoforms tagged with GFP (green) were expressed in COS-7 cells **(A)** or primary hippocampal neurons **(B)**. Cells were co-stained with 4′,6-diamidino-2-phenylindole (DAPI) to label the nuclei (blue). PSD-95 antibody was employed to distinguish hippocampal neurons. Note that Shank3b localizes in nuclei in both COS-7 cells and hippocampal neurons. *Shank3* E22: EGFP-*Shank3* exon 22 that only encodes SAM domain. Scale bar: 10 μm. Click here for file

Additional file 6: Figure S5 Shank3 isoforms are not localized in inhibitory synapses. Shank3 isoforms tagged with GFP (green) were expressed in primary hippocampal neurons and co-stained with an inhibitory post-synaptic marker Gephyrin (red). Note that there is no overlapping of Gephyrin with Shank3 isoforms. Scale bar: 10 μm. Click here for file
