# Supplementary figures and images for: Transcriptional and functional complexity of Shank3 provides a molecular framework to understand the phenotypic heterogeneity of SHANK3 causing autism and Shank3 mutant mice
Source: Mol Autism. 2014 Apr 25;5:30. doi: 10.1186/2040-2392-5-30 (PMC4113141; doi:10.1186/2040-2392-5-30)

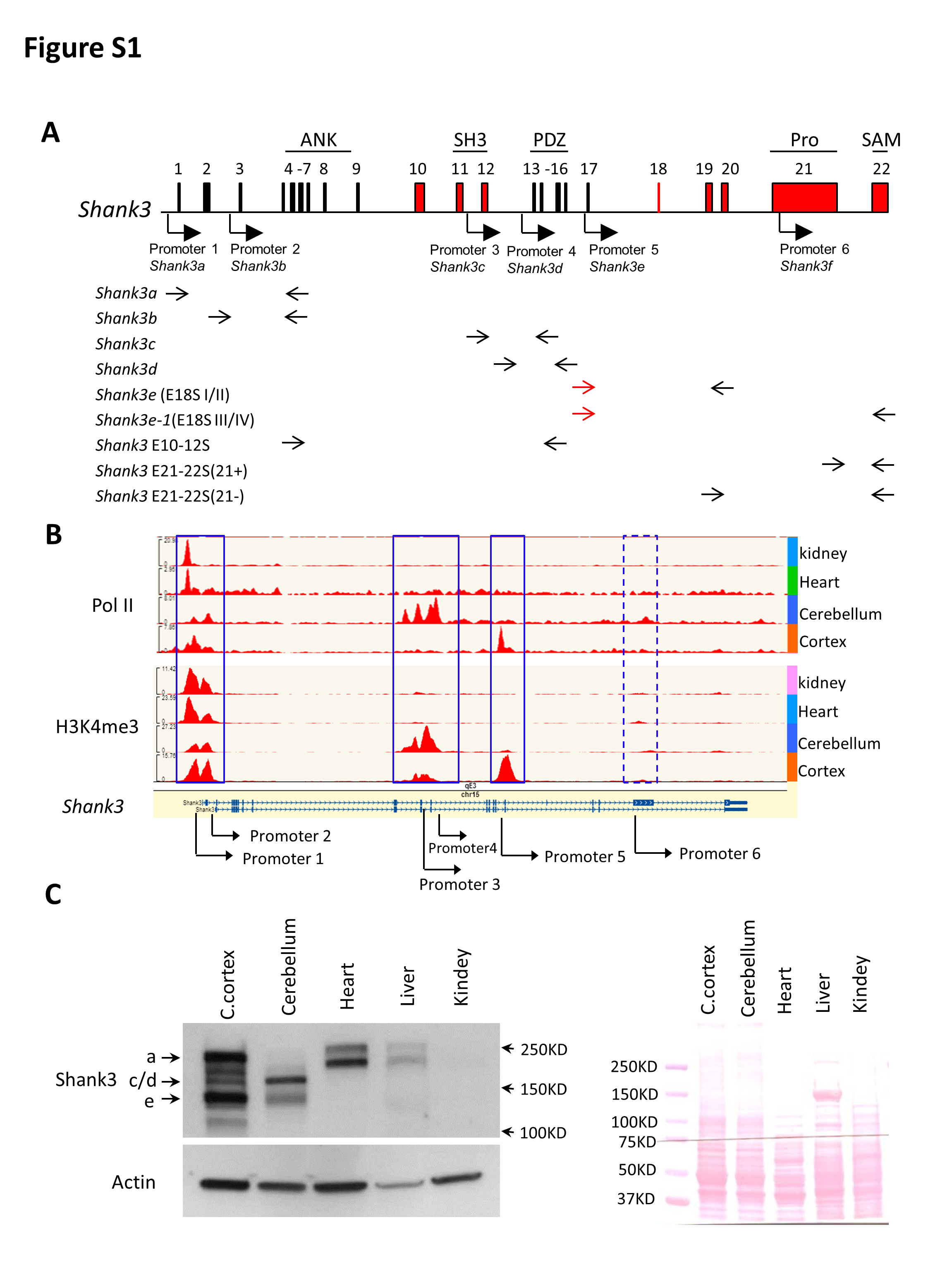

Supplement: Additional file 2: Figure S1 — Brain-specific expression of Shank3 intragenic promoters. (A) Shank3 gene structure. Intragenic promoters are shown as black arrows. Exons in red are alternatively spliced exons. In lower panel, positions of primers for Shank3 isoforms and splice variants used in this study are indicated. Note that splicing forward primer (in red) for E18S is specific for promoter 5. (B) The presence of multiple intragenic promoters supported by in silico data from ENCODE project using mouse mm10 genome assembly ( http://genomebrowser.wustl.edu/). The CHIP sequence data using antibodies against RNA polymerase II (Pol II) and trimethylated lysine 4 of histone 3 (H3K4me3), two landmarks for active promoters, displayed several corresponding peaks (blue rectangular, dash line indicates a weak promoter) near Shank3 promoters (arrows below Shank3 gene) in brain tissues but not in heart and kidney, indicating these intragenic promoters are brain specific. (C) Brain specific expression of Shank3 isoforms from intragenic promoters. Left panel: Western blot analysis using a Shank3 antibody against C-terminus of SHANK3 (sc-30193, Santa Cruz, CA, USA) revealed multiple bands across different tissues. The bands with predicted size of Shank3c, 3d, and 3e are observed in cerebral cortex and cerebellum but not in heart, liver, and kidney. Shank3b is not detectable because it does not contain the C-terminal sequence of Shank3 protein. This result supports that the intragenic promoters are brain-specific. Right panel: Ponceau S staining of the PVDF membrane on the left panel to show that an equal amount of protein (30 μg) was loaded to each lane. Note there is variation of endogenous protein composition across different tissues. [file 2040-2392-5-30-S2.tiff]

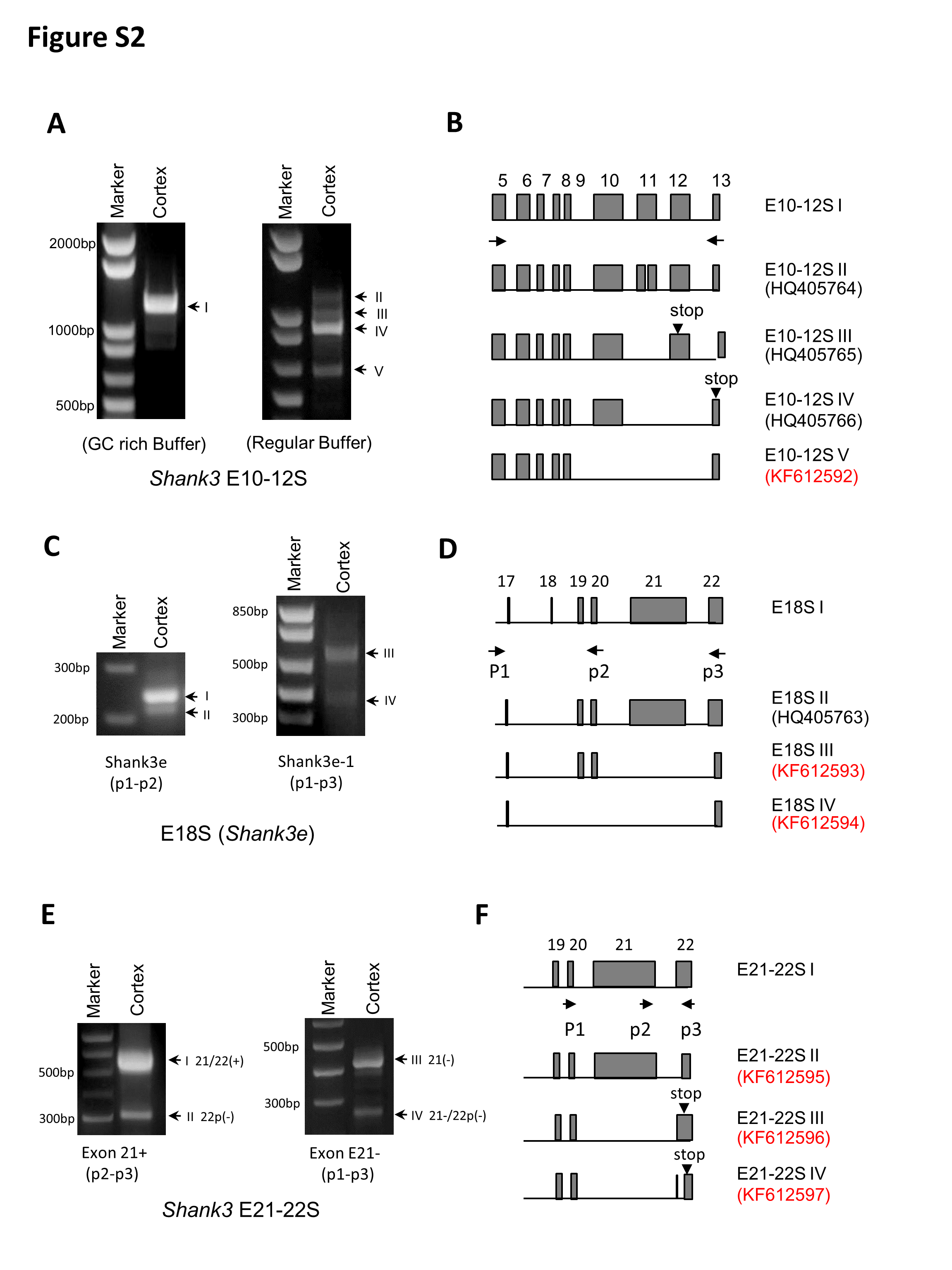

Supplement: Additional file 3: Figure S2 — Extensive splicing of Shank3 mRNAs. (A–B) Shank3 exons 10–12 spliced (E10–12S) variants. At least five different products of E10–12S are identified by RT-PCR (A) and the sequences of E10–12S variants were illustrated (B). Arrows indicate the position of primers. I: no splicing. II: exon 11 partially spliced out. III: exon 11 spliced out. IV: exons 11 to 12 spliced out. V: exons 10 to 12 spliced out. Note that regular PCR reactions yield mostly spliced variants as the full-length of this sequence without alternative splicing can only be amplified in GC-rich buffer due to the extremely high GC percentage (79%) in exon 11 (see Materials and methods). (C–D) Shank3e spliced variants (E18S). Different sets of primers yielded various PCR products (C): left panel with primers 1 and 2, right panel with primers 1 and 3. The forward primer 1 is specific for Shank3e isoforms from promoter 5. Gene structure of full-length Shank3e and its spliced variants are illustrated in (D), with arrows showing the position for primers. I: no splicing. II: exon 18 spliced out. III: exons 18, 21, and 22 (partial) spliced out. IV: exons 18 to 21 and 22 (partial) spliced out. E–F, Splicing variants of Shank3 exon 21 and exon 22 (E21–22S). The PCR products are shown in (E), with left panel using primers 2 and 3, right panel using primers 1 and 3. Gene structure of exons 19–22 and its splicing variants were illustrated in (F), with primers labeled as arrows. I: no splicing. II: exon 22 partially spliced out. III: exon 21 spliced out. IV: exon 21 and exon 22 (partial) spliced out. Gene bank accession numbers are shown in red for novel splicing variants identified by the current study. Accession numbers in black are splicing variants identified in the previous study by Wang et al. [18]. [file 2040-2392-5-30-S3.tiff]

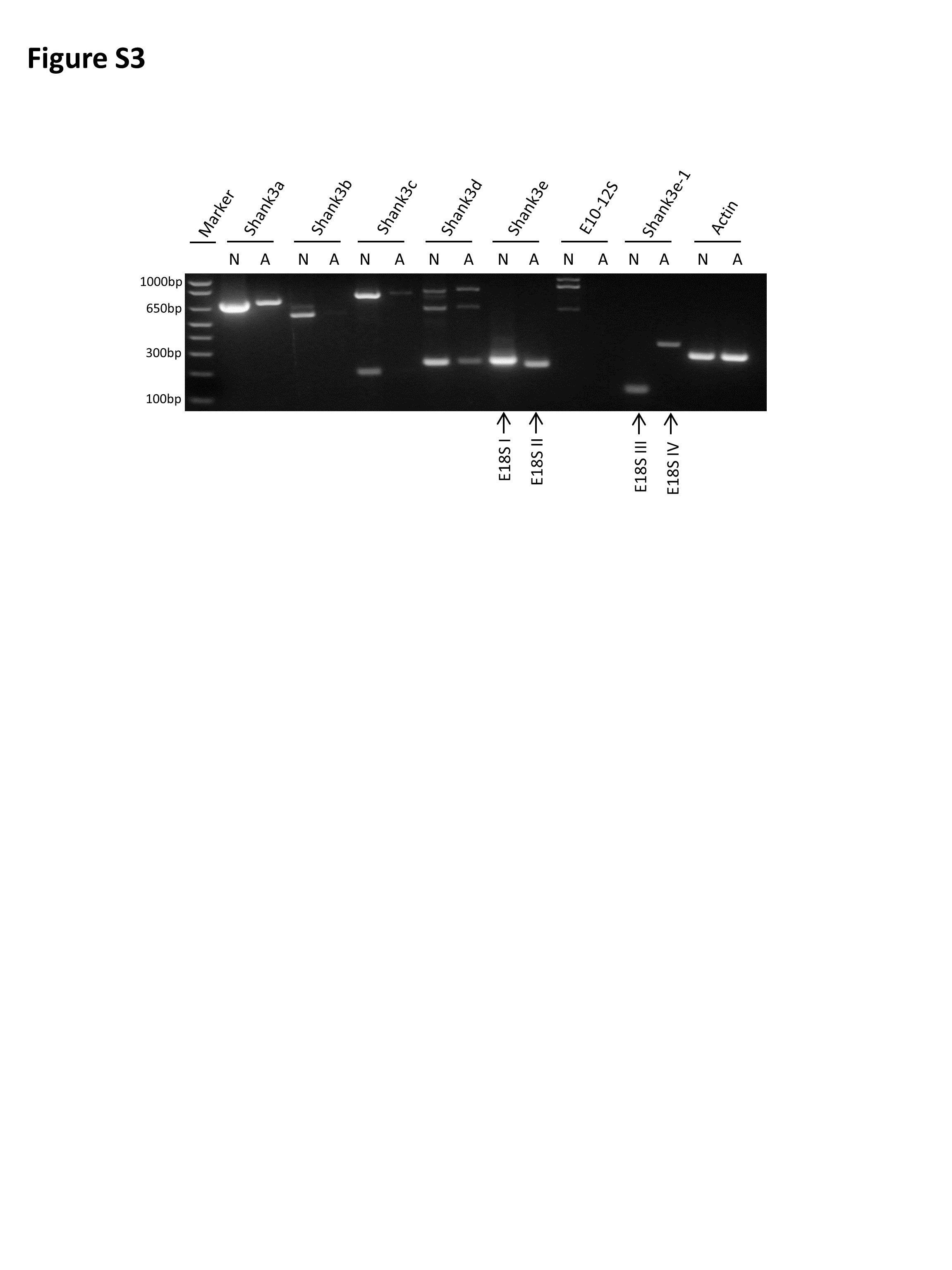

Supplement: Additional file 4: Figure S3 — Differential expression and alternative splicing of Shank3 isoforms in neurons and astrocytes. mRNAs from cultured hippocampal neurons and astrocytes were analyzed by RT-PCR. All major isoforms of Shank3 mRNAs were expressed abundantly in neurons as expected. Shank3a, 3c, 3d, 3e, and 3e-1 were low but readily detectable in astrocytes. Alternative splicing of exon 18 was mutually exclusive between cell types, with exon18 inclusion (E18S I) in neurons and exon18 exclusion (E18S II) in astrocytes. Similarly, the E18S III variant was exclusively observed in neurons while E18S IV was only found in astrocytes. N, neuron. A, astrocyte. [file 2040-2392-5-30-S4.tiff]

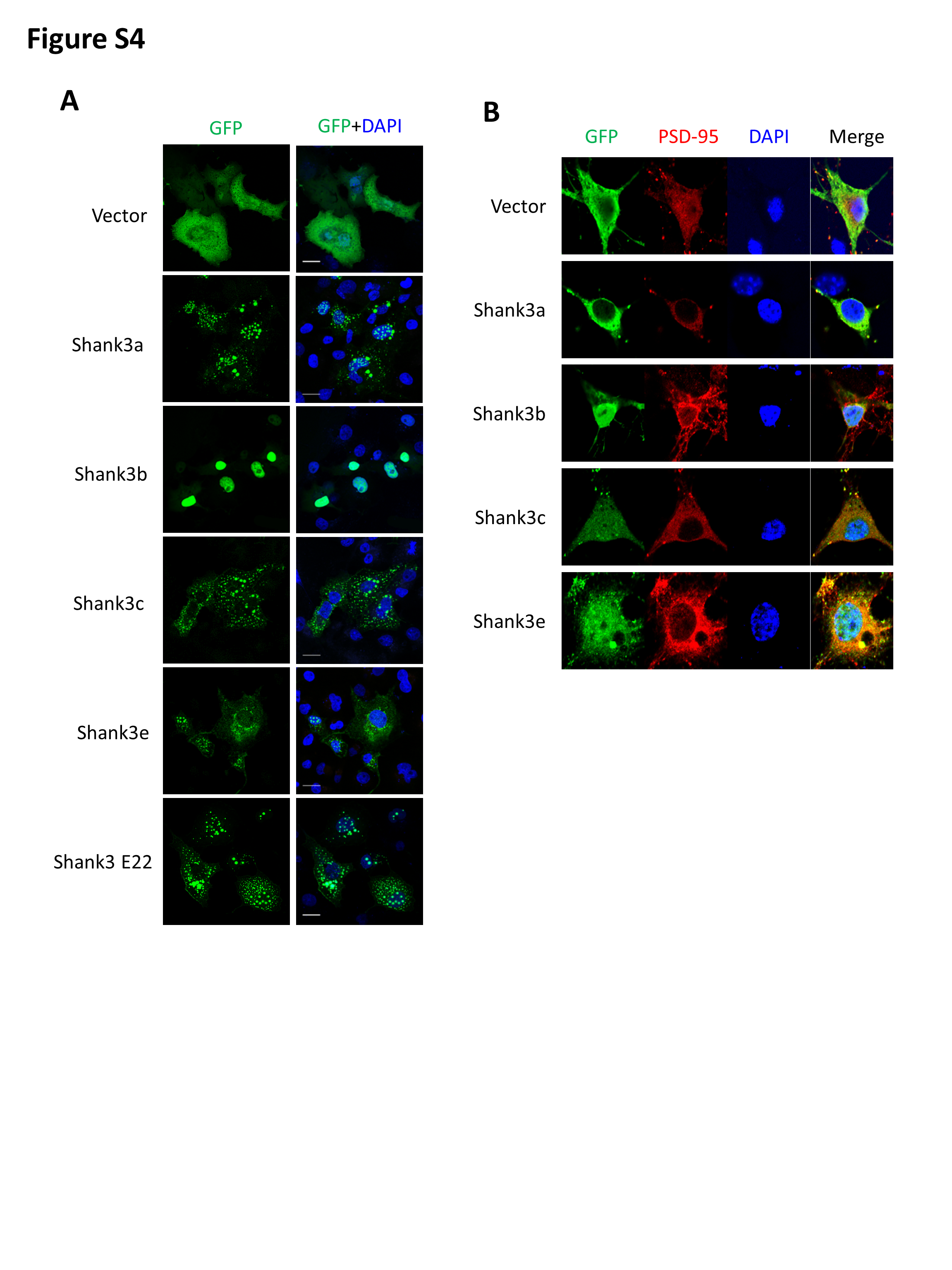

Supplement: Additional file 5: Figure S4 — Differentiated cellular distribution of Shank3 isoforms. Shank3 isoforms tagged with GFP (green) were expressed in COS-7 cells (A) or primary hippocampal neurons (B). Cells were co-stained with 4′,6-diamidino-2-phenylindole (DAPI) to label the nuclei (blue). PSD-95 antibody was employed to distinguish hippocampal neurons. Note that Shank3b localizes in nuclei in both COS-7 cells and hippocampal neurons. Shank3 E22: EGFP-Shank3 exon 22 that only encodes SAM domain. Scale bar: 10 μm. [file 2040-2392-5-30-S5.tiff]

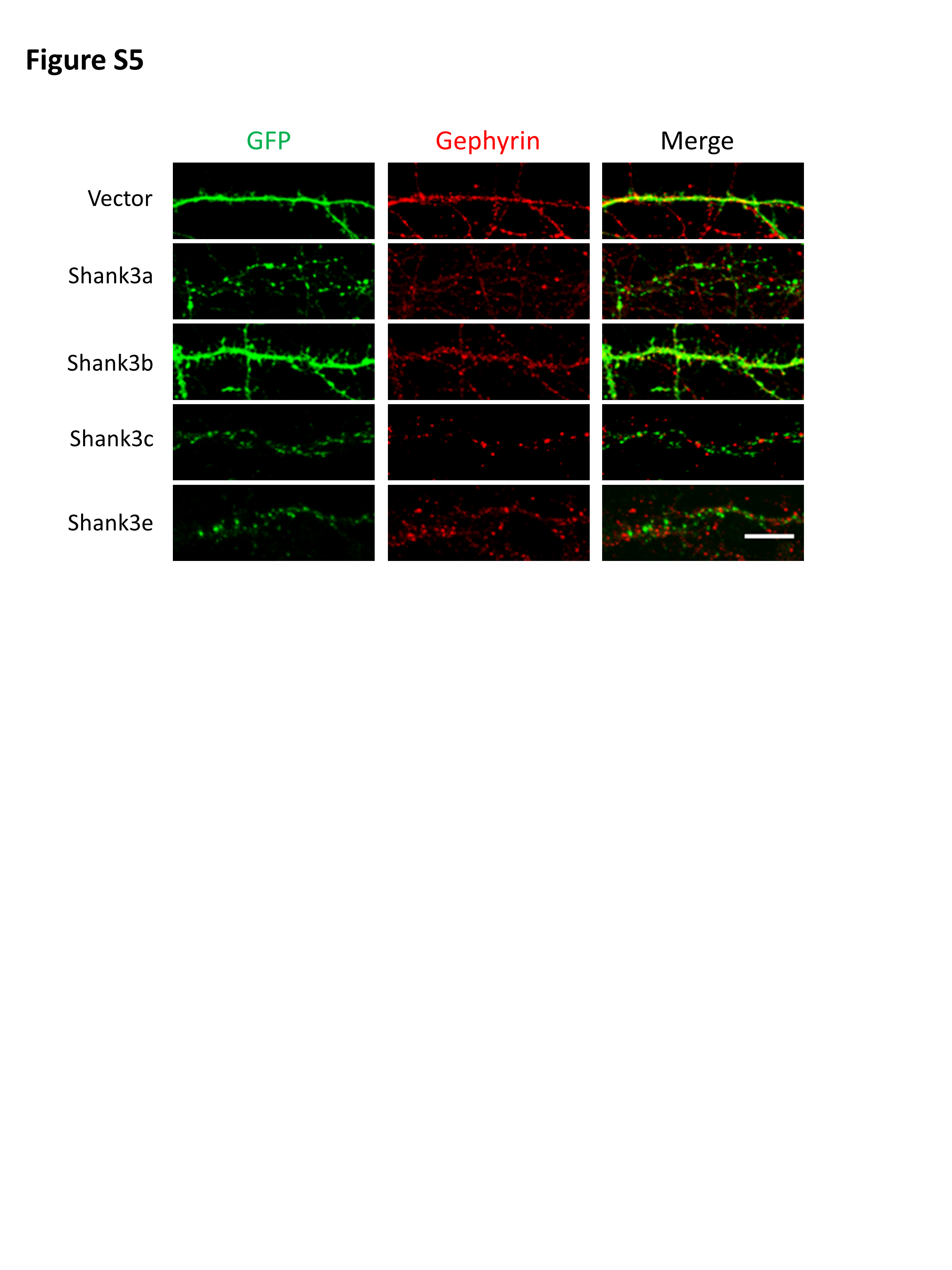

Supplement: Additional file 6: Figure S5 — Shank3 isoforms are not localized in inhibitory synapses. Shank3 isoforms tagged with GFP (green) were expressed in primary hippocampal neurons and co-stained with an inhibitory post-synaptic marker Gephyrin (red). Note that there is no overlapping of Gephyrin with Shank3 isoforms. Scale bar: 10 μm. [file 2040-2392-5-30-S6.tiff]
